# Association of vitamin D insufficiency/deficiency with thyroid artery Doppler ultrasonography in patients with Hashimoto thyroiditis

**DOI:** 10.12669/pjms.332.12566

**Published:** 2017

**Authors:** Ahmet Nalbant, Ayhan Aydin, Alper Karacan, Attila Onmez, Ali Tamer, Hakan Cinemre

**Affiliations:** 1 Dr. Ahmet Nalbant, Assistant Professor, Departments of Internal Medicine, Sakarya University School of Medicine, Sakarya, Turkey; 2 Dr. Ayhan Aydin, Internal Medicine Consultant, Departments of Internal Medicine, Sakarya University School of Medicine, Sakarya, Turkey; 3 Dr. Alper Karacan, Assistant Professor, Radiology Consultant, Departments of Radiology, Sakarya University School of Medicine, Sakarya, Turkey; 4 Dr. Attila Onmez, Assistant Professor, Departments of Internal Medicine, Düzce University School of Medicine, Turkey; 5 Prof. Dr. Ali Tamer, Departments of Internal Medicine, Sakarya University School of Medicine, Sakarya, Turkey; 6 Prof. Dr. Hakan Cinemre, Departments of Internal Medicine, Sakarya University School of Medicine, Sakarya, Turkey

**Keywords:** Vitamin D, Hashimoto thyroiditis, Doppler ultrasonography, Resistive index

## Abstract

**Background & Objective:**

During the course of the autoimmune thyroid diseases, ultrasonography change parallel to histopathology. Vitamin D is associated with autoimmune diseases and thus can affect thyroid blood flow. Our aim was to investigate the relationship between vitamin D insufficiency/deficiency and thyroid hemodynamic indices in patients with Hashimoto thyroiditis.

**Methods:**

A total of 93 patients who presented to Sakarya University Endocrinology outpatient clinic from April to September 2016 and diagnosed with Hashimoto thyroiditis were included in this study. Clinical and serologic data, thyroid antibodies and 25(OH)D3 were evaluated. Mean peak systolic velocity(mPSV), mean end-diastolic velocity (EDV), mean resistive index (RI) flows of superior and inferior thyroid arteries were measured with B-mode Doppler ultrasonography.

**Results:**

Vitamin D insufficiency/deficiency was detected in 59 (63.4%). TPO Ab and TgAb levels were found higher in patients with vitamin D insufficiency/deficiency. In the normal vitamin D group, superior thyroid artery mPSV (32.21±6.73cm/s) and EDV(13.27±2.80 cm/s) were higher than in the low vitamin D group [mPSV (28.32±8.99cm/s) and EDV(10.67±3.68 cm/s)] (P=0.034, P=0.001, respectively). Inferior thyroid artery EDV value was higher in the normal compared to the low vitamin D group (0.032). RI measured in all arteries were higher in the vitamin D insufficient/deficient group compared to the Vitamin D normal group (p=0.001).

**Conclusion:**

Vitamin-D insufficiency/deficiency has led to reduced parenchymal blood supply and increased micro-vascular resistance in Hashimoto thyroiditis patients.

## INTRODUCTION

Hashimoto thyroiditis (HT) is the most common chronic autoimmune thyroid disease characterized by painless goiter and elevated serum thyroid antibodies. Ultrasonography (USG) findings change together with the dynamic nature of the disease and histo-pathologic changes during the progression of the autoimmune disease.^1^

HT may emerge with stimulation of environmental factors in genetically susceptible individuals. Predisposing genes are human leukocyte antigen (HLA), cytotoxic T lymphocyte antigen-4 (CTLA-4), protein thyrosin phosphatase non-receptor type 22 (PTPN22) and thyroglobulin (Tg) genes.^2^ In recent years, vitamin D deficiency is reported to cause autoimmune diseases. Vitamin D receptors (VDR) were shown to be present in intestinal epithelium cells, osteoblasts, renal cells and most importantly immune system cells (T lymphocytes, monocytes, dendritic cells and also B lymphocytes).^3^ Vitamin D inhibits T lymphocyte proliferation, particularly T helper 1 (Th1) lymphocytes. It may increase T helper 2 (Th2) lymphocyte formations. Role of vitamin D in pathogenesis of HT is reduction of anti-inflammatory Th lymphocytes and elevation of inflammatory Th1 cells. T helper 17 (Th17) cells were also found to be associated with HT pathogenesis.^4^

In HT patients, diffuse, hypo-echoic, heterogeneous, hyper-vascular fields are observed on US due to the influences of autoimmunity on thyroid gland. The aim of this study was to investigate the relationship between vitamin D deficiency and hemodynamic indices on color Doppler ultrasound in HT patients.

## METHODS

A total of 93 patients who presented to Sakarya University Endocrinology outpatient clinic from April to September 2016 and diagnosed with Hashimoto thyroiditis were included in this study. Patients were divided to two groups as normal vitamin D and vitamin D insufficiency/deficiency. Clinical and serologic data, age, thyroid peroxidase auto-antibodies (TPOAb), thyro-globulin auto-antibodies (tgAb), thyroid stimulant hormone (TSH), B-mode ultrasonography, mean peak systolic velocity (mPSV), mean end-diastolic velocity (EDV), mean resistive index (RI) flows of superior thyroid artery, inferior thyroid artery measured with proper angle (45-60 °C) were measured. For all data analysis of this study, left thyroid artery measurements were used. Doppler US measurements were done by the same radiologist. Patients who had other autoimmune diseases, vitamin D and immune-suppressive drug use, malignity, who were pregnant or under 18 years were excluded from the study. Diagnosis of HT was made with clinically diagnosed hypothyroidism, presence of diffuse goiter on B-mode US, TPOAb and/or TgAb positivity.

Hormonal data, serum TSH (range, 0,35-4,94 mIU/L), free T3 (fT3) (range, 2,63-5,70 pmol/L), free T4 (fT4) levels (range 9,01-19,05 pmol/L), TgAb (cut-off level, 4.11 lU/mL), TPOAb (cut-off level, 5.61 IU/mL) were examined with automated immuno chemiluminescent assay (ICMA) kits (Abbott, İL, USA). 25 (OH) D3 levels were measured with commercial euglobulin clot lysis assay (ECLA) kit (Roche, Germany). 25 (OH) D3 status was classified as deficient (<20 ng/mL), insufficient (21-29 ng(mL) and sufficient (≥ 30 ng/mL) according to Endocrinology Society criteria.^5^

This study was approved by Sakarya University school of Medicine Ethical Committee for Clinical Research (No: 16214662/050.01.04/31).

### Statistical analysis

Data analysis was done using SPSS version 10.0 (SPSS Inc, Chicago, IL). Normally distributed parameters were expressed as mean, standard deviation (SD) and one-way ANOVAs were used for comparison of these groups. Non-normally distributed data was expressed as median, interquartile range (IR) and Kruskal-Wallis test was used for further intergroup analyses. Categorical variables were evaluated with Pearson chi-square test. A P level of <0.05 was accepted as statistically significant.

## RESULTS

A total of 93 patients were included in the study. Mean age of the patients was 36.00±12.49 years, 94.6% (n=88) of the patients were female and 5.4% (n=5) were male. Vitamin D insufficiency/deficiency was detected in 59 (63.4%) subjects, it was normal in 34 (36.6%) subjects. The general characteristics of groups according to vitamin D levels are shown in Table-I.

**Table-I:** Demographic and laboratory characteristics of vitamin D insufficient/deficient groups.

	*D vit ≥ 30 ng/mL*	*D vit< 30 ng/ mL*	*P*
Age (years), mean (SD)	38.15±14.98	34.76±10.76	0.210
TSH, IU/mL, mean (SD)	1.9±0.8	2.3±1.4	0.144
BMI, (kg/m^2^), mean, SD	27.03±4.7	25.33±3.74	0.059
TPOAb, IU/mL, median, IR	12.9 (91.52)	36.10 (235.18)	0,814
TGAb, IU/mL, median, IR	14.75 (23.00)	33.55 (81.80)	0.309

BMI:Body mass index, TPOAb:hyroperoxidase autoantibody, TgAb:thyroglobulin antibody

**Fig.1:**
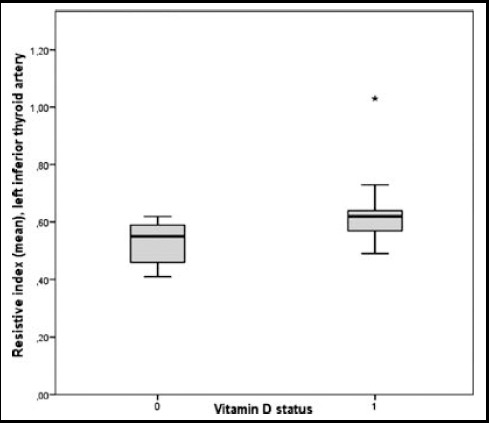
Left inferior thyroid artery, mean resistive index (ITA, RI) in vitamin-D insufficient/deficient groups.

Superior thyroid artery mPSV was found higher in normal vitamin D level group compared to low vitamin D level group (32.21±6.73 cm/s and 28.32±8.99 cm/s, respectively) (p=0.034). Superior thyroid artery EDV level was found higher in normal vitamin D level group compared to low vitamin D level group (13.27±2.80 cm/s and 10.67±3.68 cm/s, respectively) (p=0.001). Inferior thyroid artery EDV was 13.3±2.75cm/s in patients with normal vitamin D level and EDV 11.46±4.40 cm/s in patients with low vitamin D level (P=0.032). RI values measured in all arteries were found statistically significantly higher in low vitamin D level group compared to normal vitamin D level group. Superior thyroid artery RI was measured as 0.5470±0.07, inferior thyroid artery RI was measured as 0.5370±0.06 on color doppler US with subjects with normal vitamin D; superior artery RI was 0.6219±0.06 and inferior thyroid artery RI was 0.6172±0.06 in subjects with vitamin D insufficiency (p=0.001 and P=0.001) (Fig.1). Color doppler US parameters of groups are shown in Table-II.

**Table-II:** Thyroid artery color Doppler measurements in vitamin D insufficient/deficient and vitamin D normal persons.

		*D vit ≥ 30 ng/mL*	*D vit< 30 ng/ mL*	*P*
PSV(cm/s) mean, SD				
STA	sağ	30.61±6.92	30.79±9.26	0.922
	sol	32.21±6.73	28.32±8.99	0.034
ITA	sağ	31.39±7.50	31.19±9.72	0.919
	Sol	3 2.06±6.55	29.93±11.15	0.320
EDV(cm/s) mean, SD				
STA	sağ	12.61±2.87	11.39±3.81	0.115
	sol	13.27±2.80	10.67±3.68	0.001
ITA	sağ	13.24±3.13	11,86±4.23	0.106
	sol	13.3±2.75	11.46±4.40	0.032
RI mean, SD				
STA	sağ	0.5455±0.08	0.6212±0.06	<0.001
	sol	0.5470±0.07	0.6219±0.06	< 0.001
ITA	sağ	0.5282±0.07	0.6154±0.07	<0,001
	sol	0.5370±0.06	0.6172±0.06	<0.001
PI mean, SD				
STA	sağ	0.9421±0.04	0.9979±0.10	0.004
	sol	0.9403±0.03	0.9904±0.08	0.002
ITA	sağ	0.9436±0.03	0.9925±0.08	0.002
	Sol(median, IR)	0.93 (0.06)	0.98 (0.14)	<0.001

STA:Superior thyroidal artery, ITA:Inferior tiroid arter; mean peak systolic velocity:mPSV, EDV:End diastolik velocity; RI:Rezistif index, PI:Pulsatil index.

## DISCUSSION

In this study, we analyzed the influence of vitamin D level on thyroid parenchymal vascularization in patients with HT, an autoimmune disease. Blood supply is reported to increase in autoimmune thyroid diseases. Superior thyroid artery mPSV was shown to effectively discriminate the underlying cause of thyrotoxicosis.^6^ Intra-parenchymal blood supply is suggested to increase on color Doppler ultrasound examination in initial and peak stages of diffuse autoimmune thyroiditis. In this study, we showed that insufficient vitamin D level has led to decreased blood supply to thyroid in HT patients.

Vitamin D deficiency is accepted as a global health problem. Vitamin D deficiency is reported to be widespread worldwide, mainly in America, Australia, Africa and Asia.^7^ This condition may be explained with climate, geographic conditions, life style, use of sunscreens, nutrition. The relationship between low vitamin D level and autoimmune diseases is accepted by researchers. Patients with autoimmune diseases were shown to have significantly lower 25 (OH) D3 levels.^8^,^9^ A significant relationship was detected between vitamin D gene polymorphism and autoimmune thyroid diseases.^10^ Vitamin D plays an important role in regulation of T helper cell type 1 (Th1), Th2 and Th 17cells and IFN-gamma, IL-4, IL-17 secretion with its immune-modulator effect. These findings explain that low vitamin D level contributes to immune thyroid disease development.^11^-^13^ Studies have shown that 25(OH)D<20 ng/mL is a risk factor for positive thyroid antibodies (TPOAb, TgAb).^13^,^14^ In our study, TPOAb, TgAb levels were found lower in vitamin D deficient group compared to normal group in HT patients.

Blood supply of thyroid parenchyma is one of the distinctive features of thyroid diseases. Left inferior thyroid artery PSV>26.11 cm/s has 91.7% specificity for Hashimoto disease.^15^ We found inferior thyroid artery mPSV 32.06±6.55 cm/s in the group with normal vitamin D level and 29.93±11.15 cm/s in the group with low vitamin D. Inferior thyroid artery PSV values were found consistent with literature for HT. We showed that blood supply to thyroid was higher in the group with normal vitamin D. The average mPSV value of superior thyroid artery in the group with normal vitamin D level was higher than in the group with lower vitamin D level (p=0.034). Reduction of blood supply to thyroid together with vitamin D deficiency may be explained with vitamin D deficiency’s leading to higher TPOAb and TgAb levels, impaired immune-modulation, endothelial dysfunction and more severe chronic inflammation.

Studies have shown the presence of an exact intersection point in thyroid parenchymal blood supply distribution ranges in subjects with euthyroidism, thyroiditis and Graves disease.^16^ In our study, we showed the difference with superior and inferior thyroid artery EDV values with color Doppler US in subjects with normal vitamin D and HT. Resistive index was found high both in superior and inferior thyroid artery in patients with vitamin D insufficiency. Higher RI indicates blood supply to vascular bed with higher impedance.^17^ RI is positively correlated with parenchymal diseases and micro-vascular resistance. Nitric oxide was shown to be lower and endothelin was shown to be higher in patients with vitamin D insufficiency/deficiency compared to control group.^18^ Vitamin D insufficiency could have impaired parenchymal blood supply through impairing endothelial functions and leading to more parenchymal injury.

## CONCLUSION

In this study, we have showed that parenchymal blood supply of thyroid gland decreased and micro-vascular resistance increased in vitamin D insufficiency/deficiency. Increased RI is known to be seen together with carotid artery stenosis, renal transplant rejection and severe parenchymal diseases. Therefore we suggest that vitamin D insufficiency/deficiency might lead to severe parenchymal injury in HT patients. In addition, this might help in diagnosis of difficult cases by providing a certain Doppler USG pattern.

### Authors’ Contribution

**AN, AA & AO:** Did data collection and manuscript writing.

***AT & HC:*** Conceived, designed and did statistical analysis & editing of manuscript.

***AK*** Did radiological measurement of patients in radiology.

***AT:*** Did review and final approval of manuscript.

***AN:*** Takes the responsibility and is accountable for all aspects of the work in ensuring that questions related to the accuracy or integrity of any part of the work are appropriately investigated and resolved.
